# NrCAM, a neuronal system cell-adhesion molecule, is induced in papillary thyroid carcinomas

**DOI:** 10.1038/sj.bjc.6603915

**Published:** 2007-07-31

**Authors:** B Górka, J Skubis-Zegadło, M Mikula, K Bardadin, E Paliczka, B Czarnocka

**Affiliations:** 1 Department of Clinical Biochemistry and Molecular Biology, Medical Centre for Postgraduate Education, Marymoncka 99/103, 01-813 Warsaw, Poland; 2 Department of Gastroenterology and Hepatology, Medical Centre for Postgraduate Education and Maria Skłodowska-Curie Memorial Cancer Centre and Institute of Oncology, Roentgena 5, 02-781 Warsaw, Poland; 3 Department of Pathology, Medical Centre for Postgraduate Education, Ceglowska 80, 01-809 Warsaw, Poland; 4 Department of Nuclear Medicine and Endocrine Oncology, Maria Skłodowska-Curie Memorial Cancer Centre and Institute of Oncology, Wybrzeze Armii Krajowej 15, 44-101 Gliwice, Poland

**Keywords:** papillary thyroid carcinoma, neuron-glia-related cell-adhesion molecule, quantitative RT–PCR, western blot, immunohistochemistry

## Abstract

NrCAM (neuron-glia-related cell-adhesion molecule) is primarily, although not solely, expressed in the nervous system. In the present study, NrCAM expression was analysed in a series (46) of papillary thyroid carcinomas (PTCs) and paired normal tissues (NT). Quantitative reverse transcriptase (QRT)-PCR revealed that NrCAM expression was upregulated in all PTCs compared to normal thyroid, whatever the stage or size of the primary tumour. NrCAM transcript levels were 1.3- to 30.7-fold higher in PTCs than in NT. Immunohistochemistry (IHC) confirmed that the expression of NrCAM was considerably higher in tumours (score 2+/3+) than in adjacent normal paratumoural thyroid tissue. The NrCAM protein was detected in all but three (93.3%) PTC samples, and it was mainly cytoplasmic; in some cases there was additional membranous localisation – basolateral and partly apical. In the normal thyroid and tissues surrounding tumours, focal NrCAM immunolabelling was seen only in follicles containing tall cells, where staining was restricted to the apical pole of thyrocytes. Western blot analysis corroborated the QRT–PCR and IHC results, showing higher NrCAM protein levels in PTCs than in paired NT. The level of overexpression of the NrCAM mRNA in tumourous tissue appeared to be independent of the primary tumour stage (pT) or the size of the PTC. These data provide the first evidence that NrCAM is overexpressed in human PTCs at the mRNA and protein levels, whatever the tumour stage. Thus, the induction and upregulation of NrCAM expression could be implicated in the pathogenesis and behaviour of papillary thyroid cancers.

Thyroid tumours are the most frequent malignancies of the endocrine system (DeLellis and Williams, 2004). The most common type is papillary thyroid carcinoma (PTC), a well-differentiated tumour that accounts for 80–90% of all thyroid malignancies. The early stages of PTC development appear to be the consequence of abnormal genetic alteration of several proto-oncogenes, growth factor receptors, and tumour suppressor genes (Santoro *et al*, 2002; Tallini, 2002; Nikiforova *et al*, 2003). The most common genetic abnormalities associated with papillary thyroid carcinomas (PTCs) are activating mutations in *BRAF*, the gene for the B-type serine-thereonine kinase RAF, identified from 29 to 83% of patients, and RET/PTC rearrangements detected much less frequently in adult PTCs (Kimura *et al*, 2003; Namba *et al*, 2003; Soares *et al*, 2003; Kim *et al*, 2004; Santoro *et al*, 2004; Xing, 2005; Trovisco *et al*, 2006). In a variety of malignancies, tumour progression is associated with changes in cell adhesion, which affect the biological characteristics and behaviour of the cancer (Christofori, 2003; Ezzat and Asa, 2005). Cell-adhesion molecules (CAMs), belonging to the L1 family within the immunoglobulin-like superfamily, are cell-surface glycoproteins predominantly but not exclusively expressed in the peripheral and central nervous system where they mediate different aspects of neuronal tissue development (Suter *et al*, 1995; Sakurai *et al*, 2001; Falk *et al*, 2004; Zelina *et al*, 2005). These proteins contain multiple C2-type immunoglobulin (Ig)-like extracellular domains and multiple fibronectin type III (FnIII) repeats, which are connected with the cell membrane via a transmembrane domain or lipid anchor (Williams and Barclay, 1988; Gumbiner, 1996; Hortsch, 2000). The NrCAM (neuron-glia-related cell-adhesion molecule) is a 200–220 kDa transmembrane protein composed of six Ig-like domains and five FnIII repeats in the extracellular region, plus a highly conserved cytoplasmic tail (Grumet *et al*, 1991; Kayyem *et al*, 1992; Lane *et al*, 1996). In addition to the nervous system, expression of the NrCAM gene (chromosome 7q31.1–q31.2) has also been demonstrated in a variety of healthy or neoplastic tissues and cell lines including pancreatic cancer, melanoma, renal and colon carcinoma, adrenal gland, placenta, thyroid, and testis (Wang *et al*, 1998; Dhodapkar *et al*, 2001; Conacci-Sorrell *et al*, 2002a). Recent reports have linked NrCAM expression with metastatic processes in particular human cancers (Sehgal *et al*, 1998; Conacci-Sorrell *et al*, 2002b). Gene-expression profiling of PTCs using DNA microarrays showed that genes encoding adhesion molecules occur within the set of genes exhibiting differential expression in normal and cancerous thyroid tissues (Jarzab *et al*, 2005). At present, there is no information concerning the expression of NrCAM (gene or protein), its cellular localisation and distribution, or its role in the evolution of thyroid papillary carcinomas. The aim of this study was to examine the expression of this adhesion molecule by analysing both transcript and protein levels in a series of PTCs/paired normal tissues, and to investigate the association of NrCAM expression with tumour grade and stage.

## MATERIALS AND METHODS

### Patients and tissue specimens

Samples of thyroid tumour (*n*=46; 100–450 mg) were obtained from 37 women and 9 men (mean age 47.3 years; range 15–74) being treated at the Maria Sklodowska-Curie Memorial Cancer Centre and Institute of Oncology in Warsaw and Gliwice, and the Department of Pathology of the Medical Centre for Postgraduate Education in Warsaw. Histological examination was used to classify the tumours as PTCs according to WHO criteria (DeLellis and Williams, 2004). Healthy tumour-free tissues were also excised from each individual and processed in parallel. All tissue samples were immediately frozen in liquid nitrogen and stored at −80°C until they were used for quantitative reverse transcription (QRT)–PCR and protein analysis. For RNA isolation, the cancerous tissue was separated from the surrounding normal thyroid fragments following expert guidance from a pathologist. The clinicopathological features of the thyroid tumours (fresh tissues) are shown in Table 1.

For immunohistochemical analysis, archived formalin-fixed, paraffin-embedded tissues from patients who underwent surgery (total thyroidectomy) were used. Tumour staging was carried out according to the TNM (tumour, node, metastases) staging system. Fifty-three tumours, including 46 PTCs and 7 follicular adenomas (3 typical and 4 atypical) were studied. Papillary carcinomas (*n*=46) were subdivided into typical papillary carcinomas (PTC, *n*=40) and follicular variants of papillary carcinomas (FVPTC, *n*=6). A total of 28 papillary carcinomas had a papillary growth pattern and 12 had a mixed papillary-follicular growth pattern. Tumour staging and NrCAM immunoreactivity are shown in Table 2.

The local human study ethical committees approved this study, and all patients gave their informed consent.

### Quantitative (real-time) RT–PCR analysis

RNA was isolated from frozen thyroid tissues using an RNeasy Mini Kit (Qiagen, Hilden, Germany). The concentration of specific NrCAM transcript was quantified by RT (reverse transcriptase) real-time PCR. Purified total RNA (2 *μ*g) was reverse transcribed using SuperScript II RT (Invitrogen, Carlsbad, CA, USA) primed with random hexamers in a final volume of 20 *μ*l according to the manufacturer's protocol. Real-time PCR was carried out using the double-stranded DNA-specific dye SYBR Green I. The reaction mixtures contained template cDNA, 12.5 *μ*l 2X SYBR Green PCR Master Mix (PE Applied Biosystems, Foster City, CA, USA), and 50 nM primers (listed below) in a final volume of 25 *μ*l (Mikula *et al*, 2003; Skubis-Zegadło *et al*, 2005). Amplification, data acquisition, and data analysis were carried out using the GeneAmp 7000 Sequence Detection System (PE Applied Biosystems).

NrCAM (NM_005010)

Forward: 5′-TTGTGCAAAGAGGGAGCATG-3′,

Reverse: 5′-GGGCAGTTCCCTGTTGTCCT-3′,

Melt curves were generated after each run to confirm the specificity of the amplification reaction. The nature of the RT–PCR products was additionally verified by agarose gel electrophoresis. Normalisation of transcript levels to those of the housekeeping genes *GAPDH* and *β-actin* gave similar results. Polymerase chain reaction amplification efficiencies of the NrCAM gene, *GAPDH*, and *β-actin* genes were as follow: *E*_NrCAM_=1.95, *E*_GAPDH_=1.96, *E*_*β*−actin_=1.96.

Rearrangements of *RET/PTC1* and *RET/PTC3* were analysed by the real-time PCR using a specific TaqMan probe and reaction conditions as described previously (Rhoden *et al*, 2004). The reaction mixtures contained 5 *μ*l cDNA, 12.5 *μ*l 2 × TaqMan PCR Master Mix and 200 nM primers plus 100 nM of probe in a final volume of 25 *μ*l. All reactions were carried out in duplicate using the ABI 7000 system (PE Applied Biosystems). No template controls were included in each run.

### Immunohistochemistry

Sections were prepared from the blocks of tumour tissue. Slices of 3–4 *μ*m were dewaxed, deparaffinised, rehydrated, and pretreated for antigen retrieval by exposure to 0.01% protease XXIV solution (Sigma, Steinheim, Germany) at 37°C for 10 min. After endogenous peroxidase activity quenching, and blocking of non-specific binding, sections were incubated with polyclonal goat anti-NrCAM antibody (1 : 200, clone sc-18960 – epitope mapped to an internal region of NrCAM; Santa Cruz Biotechnology, Santa Cruz, CA, USA) or rabbit polyclonal antibody (1 : 600, ab24344, directed against the extracellular NrCAM domain, aa 834–856; Abcam, Cambridge, UK) overnight at 4°C in a humid chamber, followed by incubation with LSAB+detection kit reagents (DAKO A/S Glostrup, Denmark), for 15 min at room temperature. Between steps, slides were washed in Tris-buffered saline. Immunoreaction was visualised with 3, 3′-diaminobenzidine (DAB, DAKO) and nuclei were counterstained with haematoxylin. To ensure antibody specificity, consecutive sections were incubated in the absence of primary antibody or with the primary polyclonal goat antibody that had been preincubated with blocking peptide (peptide sc-18960P, Santa Cruz), and both of these controls produced no immunostaining. The staining intensity was graded using a semi-quantitative score: 0 (no staining), 1 (slight staining), 2 (moderate staining), 3 (intense staining), and the proportion of positive cells was scored as 1+ (<10% of cells), 2+ (10–50% of cells), or 3+ (>50% of cells).

### Western blot analysis

Samples of approximately 100–450 mg of frozen thyroid tissue were homogenised in an ice-cold buffer (250 mM sucrose; 20 mM Tris-HCl, pH 7.4;. 1 mM ethylene diamine tetra-acetic acid (EDTA) containing a cocktail of protease inhibitors (Roche diagnostics GmbH, Mannheim, Germany) and then centrifuged at 100 000 **g** for 1 h. The supernatants were aliquoted and stored at −80°C. The pellets, representing particulate fractions, were resuspended in 20 mM Tris-HCl, pH 7.4 containing 1 *μ*g ml^−1^ PMSF (phenyl-methyl-sulphonyl fluoride), 0.5% Nonidet-P40 and 0.1% Tween-20 and stored at −80°C. Protein concentrations in these suspensions were determined with the BCA protein assay (Pierce Chemical Co, Rockford, IL, USA) using the manufacturer's ‘microwell’ protocol.

An equal amount (50 *μ*g) of protein extract prepared from each tissue specimen was mixed with sodium dodecyl sulphate (SDS) sample buffer (0.25 mM Tris-HCl, pH 6.8; 20% glycerol; 4% SDS; 0.1% bromophenol blue), boiled for 3 min, separated on 6% SDS–polyacrylamide gel electrophoresis (PAGE) gels and electroblotted onto polyvinylidene difluoride (PVDF) membranes (Bio-Rad Laboratories, Hercules, CA, USA). After blocking, membranes were incubated overnight at 4°C with goat (1 : 2000, sc-18960, Santa Cruz) or rabbit (1 : 2000, ab24344, Abcam) polyclonal antibodies against NrCAM followed by reaction with horseradish peroxidase-conjugated affinity-purified secondary antibodies (1 : 20,000, Jackson ImmunoResearch Laboratories, West Grove, PA, USA) for 1 h at room temperature. Between steps, membranes were intensively washed in PBS-Tween20. Immunostained bands were detected by the chemiluminescent method using a SuperSignal West Pico staining kit (Pierce Chemical Co.). Western blot signals were scanned and quantified using KODAK 1D Image Analysis software (Eastman Kodak Co. Rochester, NY, USA). The size of reactive bands was determined by comparison with prestained protein markers (Bio-Rad). In a series of control blots, the primary antibodies were omitted or the goat antibody was pre-incubated with blocking peptide (sc-18960P, Santa Cruz) to confirm the specificity of the detected signals. Immunoblots were reprobed with *β*-actin antibody (1 : 10 000, mouse monoclonal antibody, Sigma) for normalisation.

The blot signal intensity obtained for NrCAM was normalised against the signal obtained for *β*-actin on the same western blot. Protein levels were expressed as fold increase with respect to normal paired thyroid tissues (=1).

### Statistical methods

Comparison of transcript levels in tumours and paired normal tissues, and the association of NrCAM transcript level or immunohistochemical expression with other tumour variables were analysed using the Mann–Whitney *U*-test. A probability value (*P*) of less than 0.05 was considered statistically significant.

## RESULTS

### Expression of NrCAM mRNA in cancerous and normal thyroid tissues

**Figure 1:**
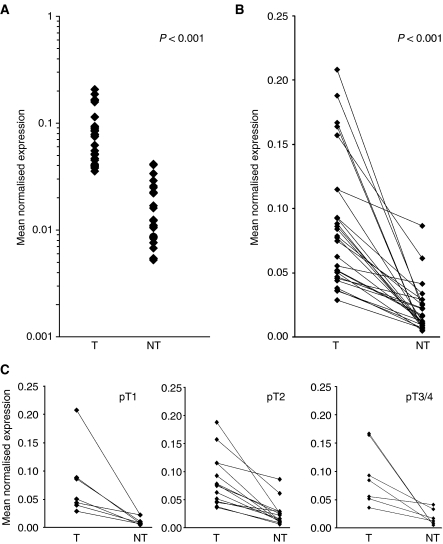
Expression of the NrCAM transcript in 46 PTCs analysed using quantitative RT–PCR. (**A**) Relative NrCAM mRNA expression in PTC (T) and normal tissues (NT); (**B**) relative expression in each PTC/normal tissue pair; (**C**) relative expression in PTCs at different stages. NrCAM, *β-actin* and *GAPDH* mRNA levels were quantified and NrCAM expression normalised against that of the two housekeeping genes. Each point represents the mean of duplicate measurements obtained for each sample in the series. Mann–Whitney *U*-test analysis was carried out and the corresponding *P* value is presented.

Quantitative (real-time) RT–PCR was used to investigate NrCAM expression in PTCs and paired normal tissues. In all cases, a single amplicon of the expected size was detected (data not shown) indicating that in both normal and cancerous thyroid tissues, NrCAM was expressed. As shown in Figure 1A, the NrCAM mRNA was clearly detected in both normal thyroid tissues and PTC samples. Expression of the NrCAM transcript was significantly higher (*P*<0.0001) in tumours (mean normalised expression 0.12±0.05), than in normal thyroid (0.021±0.019) (Figure 1B). The tumour to paired normal tissue ratio of NrCAM mRNA levels ranged from 1.3 to 30.7 (average 5.7). In 17 cases (37%), the expression was at least twofold and in 22 cases (48%) fivefold to 30-fold greater in the tumour compared with the corresponding normal tissue. The relative amount of NrCAM transcript was significantly higher whatever the tumour stage or size (Figure 1C, Table 1).

Although we observed differences in the relative amount of the NrCAM mRNA between PTCs at different stages (highest levels found in pT3/pT4N1), these were not statistically significant. There was also no association between mRNA expression and regional lymph node metastasis (data not shown).

The RET oncogene expression analysis by real-time RT–PCR revealed the presence of rearrangements in 8 out of 46 analysed samples (17.4%). Five cases (10.9%) were identified as RET/PTC1, and the other three (6.5%) as RET/PTC3 – both classic variants of PTC.

### NrCAM protein expression and cellular localisation

**Figure 2:**
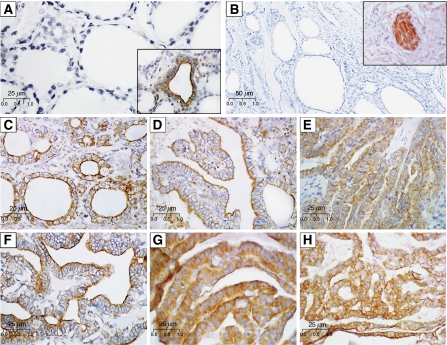
Representative immunostaining results obtained using anti-NrCAM antibodies (clone sc-18960, Santa Cruz) on sections of normal thyroid tissues and PTCs. Controls: (**A**) normal thyroid was largely unstained with some focally stained follicles (**A**-inset); (**B**) strongly stained peripheral nerves as an internal positive control (**B**-inset). PTCs: (**C**–**H**) Intense cytoplasmic and membranous staining of PTCs at different tumour stages (**C**-*pT1*, PTC with follicular and papillary pattern growth, area with follicular architecture; **D**, **E**, **F**-*pT2*, G, H-*pT3*, classic PTC with papillary pattern of growth). Original magnifications: A × 400, A inset × 400; B × 200, B inset × 400; C–H × 400.

Neuron-glia-related cell-adhesion molecule expression and tissue distribution were then evaluated by immunohistochemistry in archived paraffin-embedded samples from 46 cases of papillary carcinoma. The TNM staging, morphological subtyping of tumours and immunohistochemical analyses of NrCAM are summarised in Table 2. Representative immunostaining with specific anti-NrCAM antibodies is shown in Figure 2C–H. Most of the PTCs analysed (43/46) were positive for NrCAM expression, which was confined to tumour cells. Nineteen (41%) showed only diffuse cytoplasmic immunostaining, while 24 (52%) showed both cytoplasmic and membranous (basal and apical) staining (Figure 2C–H). Furthermore, 85% (39/46) of PTC tumours displayed intense (2+/3+) NrCAM immunoreactivity in >70% of cells. However, neither tumour stage nor size was associated with the overexpression of NrCAM (Figure 2C–H, Table 2). In follicular adenomas, focal cytoplasmic and membranous staining was observed whereas atypical adenomas were negative for NrCAM reactivity, Table 2.

The level of the NrCAM immunoreactivity was significantly higher in PTCs than in adjacent normal thyroid tissue (*P*<0.0001). The thyroid tissues next to and at a distance from the tumour were largely unstained with the exception of focally stained cells in rare follicles containing characteristic tall cells showing features of hyperactivity (Figure 2A and 2A inset). In these follicles the NrCAM staining was seen only at the apical membrane of the thyrocytes. Negative controls were consistently free of immunoreactivity (Figure 2B). Peripheral nerves used as internal positive controls showed intense NrCAM reactivity (Figure 2B inset). Overall, the presence and level of NrCAM immunostaining closely mirrored the specific mRNA abundance determined by QRT–PCR.

### Western blotting

**Figure 3:**
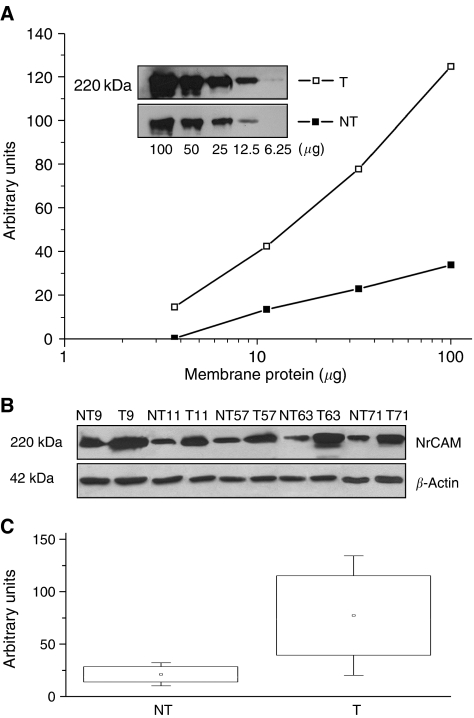
Immunoblotting of neuron-glia-related cell-adhesion molecule (NrCAM). PTC and normal thyroid tissue protein extracts were subjected to western blot analysis with antibodies against NrCAM (ab24344, Abcam) or *β*-actin. (**A**) Semi-quantitative detection of NrCAM by western blotting. Increasing amounts of pooled protein extracts prepared from 10 normal and 10 papillary carcinoma thyroid tissue samples. Chemiluminescence signals (inset) were scanned and quantified as reported in Materials and Methods. Amounts of immunoreactive NrCAM, expressed as arbitrary units, are plotted as a function of the amount of membrane protein loaded in each lane. (**B**) Comparative analyses of NrCAM content of thyroid tumours and paired normal thyroid samples. Protein extracts (50 *μ*g) from each PTC and normal paired thyroid were subjected to western blot analysis and chemiluminescence detection. *β*-Actin was then detected on the same membranes and the signals used to normalise NrCAM values. Paired samples were analysed simultaneously. Data from five representative paired tumour/normal thyroid samples are shown. (**C**) Mean NrCAM protein content in a series of PTCs and normal thyroid (±s.e.m.) expressed as arbitrary units. T-papillary thyroid carcinoma; NT-paired normal thyroid tissue.

To corroborate the QRT–PCR and immunohistochemistry results we carried out the western immunoblot analysis on 46 fresh PTC tissue samples. As shown in Figure 3A, the intensity of the NrCAM signal obtained with both specific antibodies was proportional to the protein input. Polyclonal NrCAM antibodies directed against either the extracellular or internal regions of the molecule detected a band of approximately 200–220 kDa on western blots of protein extracts from both the PTC and paired normal thyroid tissues (Figure 3A). In cancer samples, the intensity of the NrCAM protein band varied between cases; however, the reactive protein level in tumours was substantially higher than in normal thyroid tissues. This pattern was seen in all examined tumour/normal thyroid pairs. Representative western blot data for tumours and paired samples are shown in Figure 3B. On average, the level of NrCAM was 3.6-fold higher in PTC than in paired normal tissues (NT) (Figure 3C). The NrCAM protein levels determined by western blotting were consistent with those detected by immunohistochemistry (IHC), which validated the finding that NrCAM is overexpressed in human PTCs at both the mRNA and protein levels.

## DISCUSSION

Tumour progression is a multistage process in which tumour cells accumulate different abnormalities involving rearrangement, induction, or silencing of the cell-adhesion molecules (Fidler, 2002; Hirohashi and Kanai, 2003; Cavallaro and Christofori, 2004).

The neuronal cell-adhesion molecule NrCAM is mainly, but not solely, expressed in the central and peripheral nervous system. It has recently been reported that NrCAM is also expressed in a variety of other tissues, endothelial cells, certain tumour cell lines, and human cancers (Wang *et al*, 1998; Glienke *et al*, 2000; Dhodapkar *et al*, 2001; Aitkenhead *et al*, 2002; Conacci-Sorrell *et al*, 2002a). Here we present strong experimental evidence that NrCAM expression is induced in PTCs. As shown by QRT–PCR, NrCAM mRNA was present at higher levels in all PTC samples than in normal thyroid tissues, and its overexpression occurs at the transcriptional level. Because NrCAM overexpression was seen in all examined cases, we assessed whether it could be associated with tumour progression or metastasis. The NrCAM upregulation was found in all PTCs so its presence was apparently not related to their differentiation grade or stage. We did observe that tumours with more aggressive clinical behaviour (eg, pT4N1) tended to express higher levels of NrCAM, suggesting possible involvement of this molecule in regional spread – a characteristic feature of papillary carcinomas – but these differences were not statistically significant. Therefore, it seems that the NrCAM induction and upregulation in PTCs is also not related to nodal metastasis.

Immunohistochemistry verified the QRT–PCR results at the protein level. The immunostaining pattern of papillary tumours, peritumoural, and normal thyroid tissues unequivocally demonstrated that NrCAM is expressed by tumour cells and not merely by nervous components of the thyroid gland. Our observation that focal immunostaining was rare in the normal thyroid, while such staining was common in tumours, indicated that elevated NrCAM expression is specific for tumour cells and represents a true tumour-associated abnormality. In the vast majority of PTCs, NrCAM protein expression was high, as estimated by the intensity of staining. Moreover, the changes in the level of expression reflected by altered immunoreactivity were consistent with those detected by QRT–PCR. Strong NrCAM immunolabelling was found irrespective of the tumour stage or size, including clinically quiescent micro-carcinomas.

Because primary tumours contain both cancer cells and a variety of stroma cells, it was important to carefully examine the localisation of the NrCAM protein. In normal thyroid, NrCAM was detected only in rare follicles containing tall cells showing features of hyperactivity, and in these cells the protein was located exclusively at the apical pole of thyrocytes. In contrast, a diffuse cytoplasmic pattern of NrCAM distribution was seen in all analysed PTCs. In some tumours, membranous, both apical and basal, NrCAM localisation was also observed.

The quantitative RT–PCR and IHC results were corroborated by western blot analysis. In all cases, the levels of immunoreactive NrCAM protein were higher in tumours than in normal paired tissues. In summary, we found induction and overexpression of NrCAM in tumours arising from cells that very rarely express this cell-adhesion molecule in normal tissue.

The biological basis of NrCAM induction, overexpression, intracellular, and basal localisation is as yet unclear. Four genetic defects, at the somatic level, are associated with papillary cancers of the thyroid: chromosomal recombination events affecting RET and TRKA, and activating mutations of *RAS* and BRAF. The RET *and* TRKA genes encode membrane tyrosine kinase receptors for neural growth factors: RET is one of the receptors for glial cell line-derived neurotrophic factor, whereas TRKA is a receptor for nerve growth factor (Klein *et al*, 1991; Airaksinen *et al*, 1999). One of the mechanisms involved in NrCAM induction seems to be the RET chromosomal rearrangement. Activation of the RET gene at its chromosomal locus occurs in from about 20% to more than 40% of sporadic PTCs, including micro-carcinomas, and is thus an early event in thyroid carcinogenesis (Nikiforov, 2002; Santoro *et al*, 2004). It has recently been shown that artificially raised expression of RET/PTC1 – one of two most prevalent RET/PTC variants found in the vast majority of PTCs – in normal human thyrocytes, directly induces many inflammatory and tumour-invasion genes including the NrCAM gene (Borello *et al*, 2005). In this study, we found similar NrCAM upregulation at both the transcriptional and protein levels in a large number of PTC thyroid samples. Thus, it is conceivable that in PTC cells, RET-derived chimeras might trigger NrCAM induction and upregulation. In the present study RET/PTC1 and RET/PTC3, the most frequent rearrangements detected in papillary carcinomas, occurred with a similar frequency: RET/PTC1 was present in five cases of PTC (10.9%) and RET/PTC3 in three cases (6.5%). These frequencies are similar to those reported previously (Santoro *et al*, 2002). However, taking into account the high frequency of NrCAM overexpression we observed, and the prevalence of RET/PTC, it is likely that NrCAM induction in PTCs is an event only partly dependent on the RET/PTC rearrangement, and other factors are likely to be involved. The most common genetic abnormality identified from 29 to 83% of PTCs is a somatic point mutation in the BRAF gene leading to a V600E substitution (Xing, 2005). The occurrence of the BRAF^V600E^ mutation is associated with particular PTC histotypes, being much more common in the classic type of PTCs than in FVPTC where another mutation, BRAFK601E, is found with an overall frequency of up to 12% (Soares *et al*, 2003; Trovisco *et al*, 2004, 2006; Xing, 2005). Among the PTCs examined in the present study there were only four cases of the follicular variant of PTC, and in these carcinomas the NrCAM transcript was overexpressed in comparison with normal thyroid. In the series of archived cases there were six FVPTCs, all showing NrCAM protein overexpression. Although we did not analyse the BRAF^V600E^ mutation, the fact that two-thirds of PTCs show this genetic abnormality and in view of high prevalence of NrCAM mRNA overexpression found in this series of PTCs (all cases), it may be speculated that constitutive signalling along the RET–RAS–BRAF–MAPK pathway may initiate NrCAM neoexpression in PTCs. Further studies are required to determine the relationship between PTC-associated genetic abnormalities and the induction of NrCAM expression.

Many different cell-adhesion molecules are implicated in human carcinogenesis. Neuronal cell-adhesion molecules act in cell adhesion, although their effects are also linked with different recognition processes and signal transduction pathways regulating cell differentiation, proliferation, or migration (Gumbiner, 1996; Cavallaro and Christofori, 2004). Several lines of evidence indicate that expression of NrCAM and other members of the L1-CAM family might be important in the biological behaviour of human tumours (Wang *et al*, 1998; Dhodapkar *et al*, 2001; Fogel *et al*, 2003; Allory *et al*, 2005; Euer *et al*, 2005). Previous studies have shown that NrCAM is a target gene of *β*-catenin signalling in human melanoma, and colon carcinoma cell lines and tissues (Conacci-Sorrell *et al*, 2002b). The induction of NrCAM transcription by *β*- or *γ*-catenin plays a role in melanoma and colon carcinogenesis, most likely by promoting cell growth and motility (Conacci-Sorrell *et al*, 2002b). Furthermore, the NrCAM ectodomain was found to be shed from the cell surface, and could activate various signalling pathways, enhance cell motility and confer tumourigenesis in mice (Conacci-Sorrell *et al*, 2005). Moreover, suppression of NrCAM expression by siRNA in melanoma cells inhibited the adhesive and tumourigenic ability of these cells (Conacci-Sorrell *et al*, 2005). Increased NrCAM levels were found in well- or moderately differentiated pancreatic cancers, whereas this protein was reduced or absent in most poorly differentiated tumours, suggesting that differential NrCAM expression may be involved in the pathogenesis and invasive/metastatic behaviour of pancreatic tumours (Dhodapkar *et al*, 2001). Recently, the neoexpression of the coreceptor of the active form of TRKA, p75 neurotrophin receptor (p75NTR), was demonstrated in a series of PTCs in comparison with normal thyroid (Rocha *et al*, 2006). The expression of this neuronal protein was found only in PTC, especially in typical papillary carcinomas. Immunohistochemical examination of p75NTR expression with analysis of BRAF^V600E^ in typical PTCs did not show a significant correlation between this mutation and immunopositivity, although an association between the presence of BRAF^V600E^ and apical protein localisation was found. Despite the fact that we have no data concerning the frequency of BRAF^V600E^ in the present series of tumours, NrCAM transcript expression was significantly higher in every analysed cancer. The relevance of increased NrCAM expression in PTCs is unknown at present. Its induction and overexpression in tumour cells regardless of the primary tumour stage suggests that this is an early event during PTC development.

Our results support the suggestion that NrCAM is one of the several adhesion molecules that are involved in thyroid carcinogenesis (Brabant *et al*, 1995). However, further studies are needed to fully investigate the role of the NrCAM in the biology of thyroid epithelial papillary carcinomas.

## Figures and Tables

**Table 1:** Tumour to normal tissue mRNA ratio in relation to clinicopathologic features of the papillary tumours

**Stage**	**No.**	**Patient**	**Sex/Age**	**Histology**	**TNM**	**T/N**
T1	1	GD	F/36	PTC	T1bN1bMo	9.28
	2	MT	F/44	PTC	T1bNoMo	11.49
	3	OJ	F/51	PTC	T1N1Mo	2.43
	4	MT	M/43	PTC	T1NoMo	8.06
	5	KM	F/51	PTC	T1NoMo	9.56
	6	ND	F/32	PTC	T1bNxMx	2.10
	7	KB	F/44	PTC	T1NxMo	3.13
	8	PM	F/45	PTC	T1aNxMx	1.27
	9	TM	F/46	PTC	T1aNoMo	5.10
						
T2	10	ZM	F/35	PTC	T2bN1Mo	2.55
	11	KB	F/58	PTC	T2NoMx	2.25
	12	PB	F/	PTC	T2bNoMo	5.56
	13	SM	M/	PTC	T2N1M1	5.16
	14	MM	F/17	PTC	T2N1M1	5.13
	15	JS	M/52	PTC	T2N1Mo	2.12
	16	KK	F/9	FVPTC	T2N1Mo	2.01
	17	SE	F/43	PTC	T2N1Mo	7.32
	18	RT	F/48	FVPTC	T2NoMo	1.12
	19	KE	F/23	PTC	T2NoMo	3.16
	20	CA	F/	PTC	T2NoMo	1.33
	21	SP	F/15	PTC	T2NoMo	5.08
	22	MZ	F/60	PTC	T2NoMo	2.96
	23	JM	F/69	PTC	T2NoMo	9.27
	24	KJ	F/46	PTC	T2NoMo	6.87
	25	SH	F/32	PTC	T2NoMo	8.41
	26	JJ	F/64	PTC	T2NoMo	3.08
	27	WB	F/50	PTC	T2NoMx	1.12
	28	DJ	F/57	PTC	T2NxMo	7.29
	29	BT	F/59	PTC	T2N1Mo	2.93
	30	JM	M/27	PTC	T2NoMo	2.59
	31	ZJ	F/60	PTC	T2NoMo	2.64
	32	CT	F/	PTC	T2NoMo	1.31
						
T3/T4	33	CT	F/	PTC	T3N1Mo	3.05
	34	SI	F/18	PTC	T3bN1Mo	5.11
	35	HH	F/45	FVPTC	T4bN1bMx	5.03
	36	NK	F/58	PTC	T4bN1M1	1.15
	37	DB	F/60	PTC	T4bN1Mo	30.37
	38	ZG	F/71	PTC	T4bNoMo	2.92
	39	ZJ	F/69	FVPTC	T4bNxMo	5.91
	40	WK	F/64	PTC	T4N1M1	5.78
	41	PM	F/74	PTC	T4N1Mo	1.35
	42	CE	F/68	PTC	T4NoMo	9.68
	43	MJ	F/49	PTC	T4NoMo	1.33
	44	KD	M/16	PTC	T4N1bM1	2.01
	45	SE	M/	PTC	T3N1M1	15.82
	46	ND	F/49	PTC	TxNoMo	30.75

Abbreviation: TNM=tumour, node, metastases.

**Table 2:** NrCAM immunoreactivity in relation to papillary carcinomas stage and variant

			**NrCAM immunostaining**			
***T***	**Subtype *n*=53**	**Node metastases**	**>50% (3+/2+)**	**11-50% (2+/1+)**	**<10% (1+)**	**0**
	FA (3)				3	
	AA (4)					4
T1	PTC (3)			2	1	
	FVPTC					
						
T2	PTC (36)	1	18	12	5	1
	FVPTC(4)		1	3		
						
T3	PTC (1)		1			
	FVPTC(2)	1		1		1

Abbreviation: NrCAM**=**neuron-glia-related cell-adhesion molecule; FA=follicular adenoma; AA=atypical adenoma, PTC=papillary thyroid carcinoma; FVPTC=follicular variants of papillary carcinoma.
